# Unanticipated pathological clearance in two cases of clinical T4b dMMR/MSI-h advanced colorectal cancer: the potential of immune checkpoint inhibitors despite positive positron-emission tomography results

**DOI:** 10.1186/s40792-024-01894-x

**Published:** 2024-05-01

**Authors:** Daigaku Nakamura, Takeshi Yanagita, Yoshiaki Fujii, Kaori Watanabe, Takuya Suzuki, Hajime Ushigome, Ruriko Nishigaki, Naomi Sugimura, Mamoru Tanaka, Ryo Ogawa, Hiroki Takahashi, Takaya Shimura, Yuji Hotta, Yoichi Matsuo, Masahiro Kondo, Yoko Furukawa-Hibi, Shuji Takiguchi

**Affiliations:** 1 Department of Pharmacy, Nagoya City University Hospital, Kawasumi 1, Mizuho-Cho, Mizuho-Ku, Nagoya, 467-8602 Japan; 2 Department of Gastroenterological Surgery, Nagoya City University Graduate School of Medical Sciences, 1-Kawasumi, Mizuho-Cho, Mizuho-Ku, Nagoya City, Aichi, 467-8601 Japan; 3 Department of Clinical Pharmaceutics, Nagoya City University Graduate School of Medical Sciences, Kawasumi 1, Mizuho-Cho, Mizuho-Ku, Nagoya, 467-8601 Japan; 4 Department of Gastroenterology and Metabolism, Nagoya City University Graduate School of Medical Sciences, Kawasumi 1, Mizuho-Cho, Mizuho-Ku, Nagoya, 467-8601 Japan

**Keywords:** Immunotherapy, Neoadjuvant, Colorectal cancer, Case report, Mismatch repair deficient, Microsatellite instability, Positive positron emission tomography, Immune checkpoint inhibitor

## Abstract

**Background:**

The standard treatment for colorectal cancer consists of surgery and chemotherapy, which can be combined to improve outcomes. Immune checkpoint inhibitors (ICI) are a significant advancement in the standard treatment of metastatic, unresectable colorectal cancer with deficient mismatch repair (dMMR). However, limited data are available about the use of ICI in the neoadjuvant and conversion settings. Here, we present two cases treated with ICI.

**Case presentation:**

Case 1: A 75-year-old male with a large, borderline resectable rectal cancer diagnosed as cT4bN1bM0 who underwent neoadjuvant chemotherapy, followed by combination ICI consisting of ipilimumab and nivolumab. After four courses of ICI, the tumor significantly shrank, but positron emission tomography still showed a positive result and R0 resection was performed. Pathological analysis revealed no residual cancer cells. The patient has been monitored without adjuvant chemotherapy, and no recurrences have occurred after one year. Case 2: A 60-year-old male with locally advanced sigmoid colon cancer who received neoadjuvant treatment with pembrolizumab. The tumor partially shrank after three courses, and continued pembrolizumab monotherapy resulted in further tumor shrinkage which still showed positive positron emission tomography. Curative sigmoidectomy with partial resection of the ileum and bladder was performed, and the pathological outcome was pCR. There was no viable tumor in the specimen. The patient has been monitored without adjuvant chemotherapy for six months, and no recurrence has been observed.

**Conclusions:**

The present study reports two cases, including a large, borderline resectable rectal cancer after failure of chemotherapy followed by combination treatment with nivolumab and ipilimumab and one case of sigmoid colon cancer after pembrolizumab treatment, which resulted in pathological complete response. However, it remains unknown whether ICI therapy can replace surgery or diminish the optimal extent of resection, or whether adjuvant chemotherapy is needed after surgery in the case of achieving pCR after ICI therapy. Overall, this case report suggests that ICI before colorectal surgery can be effective and potentially a ‘watch-and-wait” strategy could be used for cases in which ICI is effective.

## Background

There has been a rapid improvement in treatment options for locally advanced colorectal cancer in the past years. This has resulted in revisions of guidelines for preoperative and postoperative chemotherapy and/or chemoradiation therapy [1–6]. In particular, chemotherapy for DNA mismatch repair-deficient/microsatellite instability-high (dMMR/ MSI-h) cases has been reported to result in favorable responses. Recently, several promising case series have reported remarkable effects of anti-PD-1/PDL-1 inhibitors for patients with dMMR/ MSI-h colorectal cancer [7–19]. Neoadjuvant immunotherapy was preferably recommended to treat T3/T4 colorectal cancer with dMMR/ MSI-h in the National Comprehensive Cancer Network (NCCN) guidelines version 2.2023 [20, 21], whereas no recommendation were made on the use of anti-PD-1/PDL1 inhibitors in the European Society for Medical Oncology (ESMO) guidelines [22]. The Japanese Society for Cancer of the Colon and Rectum (JSCCR) guidelines from 2019 for the treatment of colorectal cancer only recommend that patients with unresectable colorectal cancer be treated with pembrolizumab and no recommendations were made for anti-PD-1/PDL1 inhibitors as a neoadjuvant therapy [23].

Here, we describe two cases with dMMR/ MSI-h marginally resectable colorectal cancer in which the tumor invading another organ (cT4) shrank dramatically after immunotherapy, even though there was still fluorine-18-fluorodeoxy-d-glucose (FDG) uptake. Radical resection was performed after ICI treatment, resulting in pathological complete response.

## Case presentation

### Case 1

**Fig. 1:**
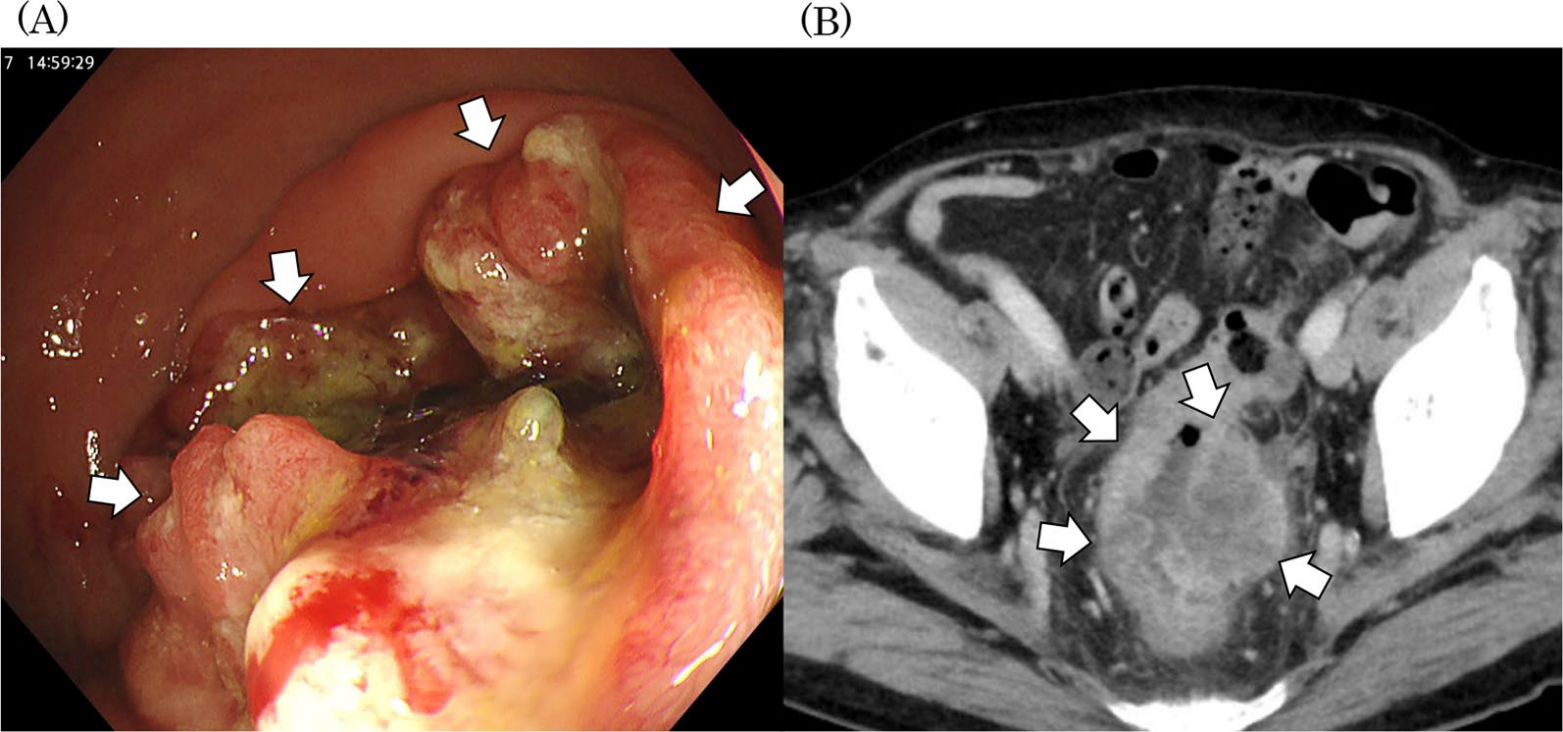
Baseline imaging for Case 1. **A** Endoscopic imaging showing a rectal tumor 7 cm from the anal verge. **B** Computerized tomography (CT) scan staging a locally advanced rectal cancer (cT4aN1bM0). Arrows identify the tumor

**Fig. 2:**
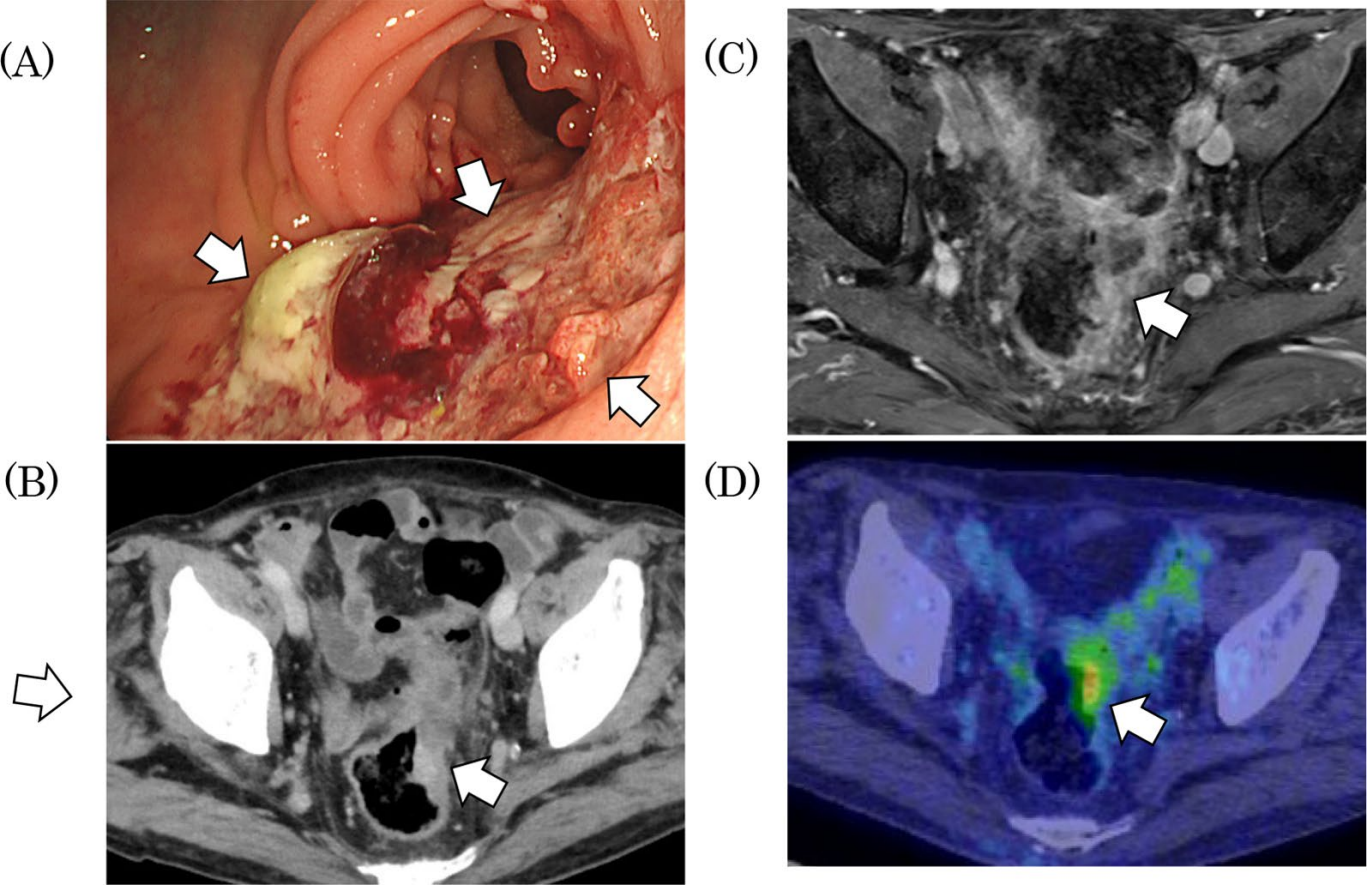
Imaging conducted after treatment with neoadjuvant mFOLFOX6 followed by ipilimumab and nivolumab in Case 1. **A** Endoscopic imaging showing a sigmoid colon cancer turned ulcer. **B** CT scan, after ipilimumab and nivolumab, showing the tumor’s notable shrinkage. **C** Magnetic resonance imaging (MRI) after ipilimumab and nivolumab therapy, showing the tumor’s notable shrinking. **D** Positron emission tomography (PET)–CT after ipilimumab and nivolumab therapy, showing the existence of tumor hot spots. Arrows identify the tumor

A 75-year-old male was diagnosed with a large, borderline resectable, rectal cancer with dMMR/ MSI-h. Imaging studies revealed a suspected invasion of the tumor to the left peritoneum and hypogastric nerve. The patient was staged as having clinical T4bN1bM0, and the main tumor was located about 7 cm from the anal verge (Fig. 1). According to the JSCCR 2019 guidelines for the treatment of colorectal cancer, we considered upfront surgical resection as a feasible option, but the size and circumferential invasion would make the surgery highly invasive, while being potentially noncurative. Therefore, we decided to administer neoadjuvant treatment. Neoadjuvant chemotherapy with oxaliplatin, leucovorin, and fluorouracil (mFOLFOX6), a standard therapy recommended in the JSCCR 2019 guidelines [23], was administrated for four cycles. In response to this, a follow-up MRI scan revealed stable disease. We concluded it would still be challenging to perform R0 resection. Therefore, we then decided to treat with the combination ipilimumab (1 mg/kg) and nivolumab (360 mg) every three weeks, based on the high-MSI status of the tumor. After four courses (approximately three months) of the ICI, the tumor had shrunk extensively. However, positron emission tomography (PET)–CT scan showed FDG uptake at the tumor site, which led us to assume the tumor was resectable with a free surgical margin if a combined resection of the left hypogastric nerve and pre-hypogastric nerve fascia was performed (Fig. 2). Robotic low anterior resection was performed two weeks after the last ICI dose, in which the left hypogastric nerve was resected. We detected no residual cancer cell in the resected specimen, and we concluded that pathologic complete response (pCR) was obtained. The patient has been monitored without adjuvant chemotherapy. One year has passed since the R0 resection was performed and no signs of recurrence have been observed since.

### Case 2

**Fig. 3:**
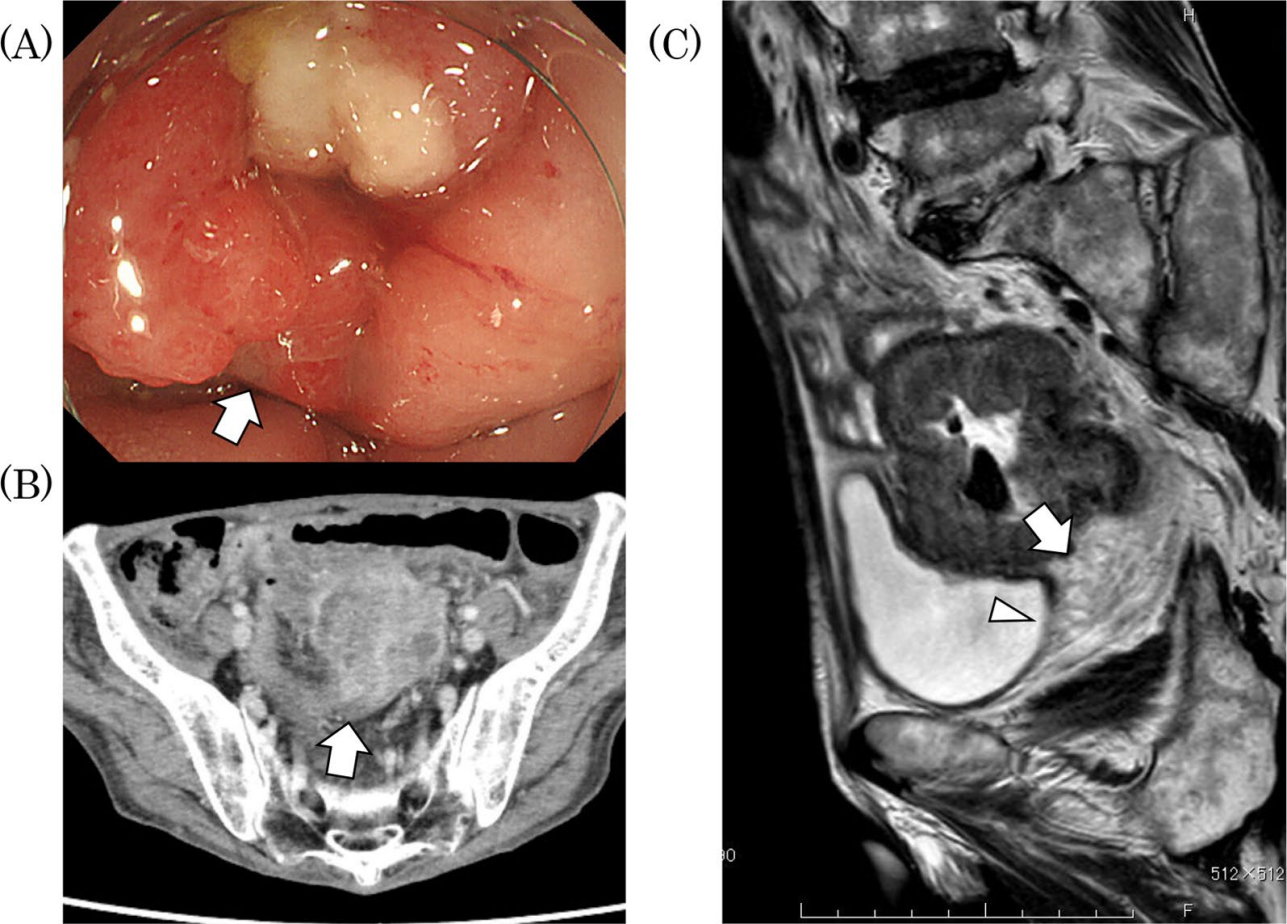
Baseline imaging for Case 2. **A** Endoscopic imaging showing a sigmoid colon tumor. **B** Computerized tomography (CT) scan staging a locally advanced sigmoid colon cancer (cT4bN1bM0). **C** Magnetic resonance imaging (MRI) showing a suspected tumoral invasion to the bladder (arrowhead). Arrows identify the tumor

**Fig. 4:**
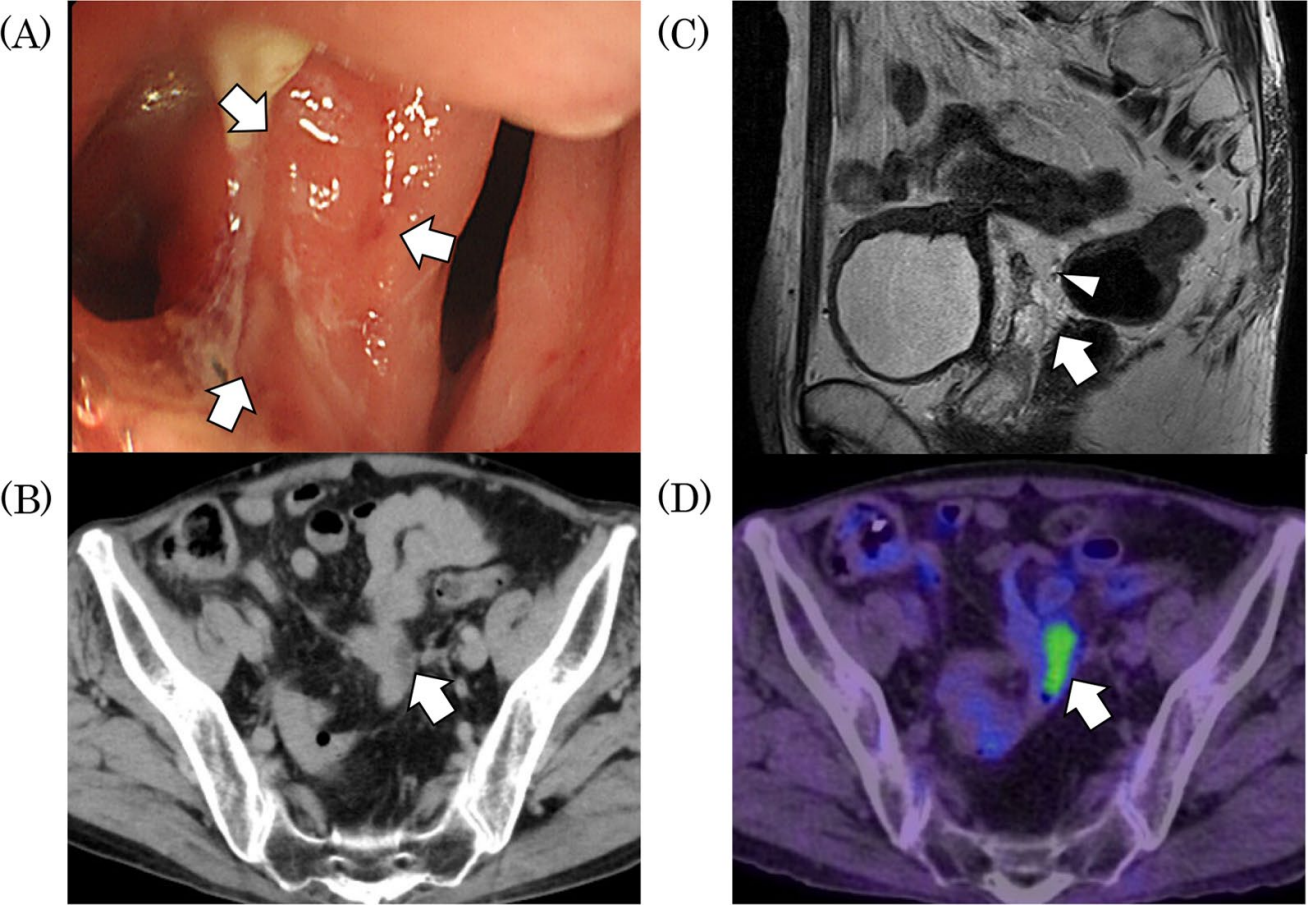
Imaging after treatment with pembrolizumab in Case 2. **A** Endoscopic imaging showing a sigmoid colon tumor turned fistula. **B** CT scan, after ipilimumab and nivolumab treatment, showing the tumor’s notable shrinking. **C** Magnetic resonance imaging (MRI) showing the fistula (arrowhead) between the sigmoid colon cancer and ileum. **D** PET–CT after ipilimumab and nivolumab treatment showing a low signal on the borderline between the sigmoid colon cancer and the bladder. Arrows identify the tumor

A 60-year-old male had been diagnosed with locally advanced, left-sided sigmoid colon cancer with invasion into the ileum and bladder. A biopsy sample histologically showed the tumor was dMMR/MSI-high. The patient was staged as having clinical T4bN1bM0, and the main tumor was located about 15 cm from the anal verge (Fig. 3). Total pelvic exenteration and partial resection of the small intestine was necessary for R0 resection due to the invasion into the ileum and bladder. Therefore, we decided to administer neoadjuvant treatment with pembrolizumab (200 mg) monotherapy every three weeks after ileostomy had been performed because of small bowel obstruction. After three courses of pembrolizumab, the tumor had partly shrunk as revealed by a follow-up CT scan. Based on these results, we decided to continue pembrolizumab monotherapy. The patient received eight courses of pembrolizumab (for six months), resulting in further shrinkage of the tumor as observed by follow-up colonoscopy, barium enema exam, MRI, and CT scans. The MRI showed fistula formation between the ileum and primary tumor site due to tumor regression and a decrease in the extent of bladder invasion. On the other hand, a PET–CT scan showed a small hot spot remained at the sigmoid colon cancer invading to the ileum. Colonoscopy with biopsy of the suspected tumor site was performed, which was negative for malignancy (Fig. 4). Finally, we performed curable sigmoidectomy with partial resection of the ileum and bladder. The pathological outcome was pCR, and there was no viable tumor left in the resected specimen. After surgery, the patient was carefully monitored for recurrence in the outpatient setting without adjuvant chemotherapy for six months.

## Discussion

There are differences in guidelines on recommendations regarding preoperative treatment for patients with dMMR/ MSI-h colorectal cancer. The NCCN guidelines suggest that treatment options for these patients include neoadjuvant therapy [20, 21], whereas the ESMO [22], and JSCCR guidelines [23] do not discuss such therapies.

The two cases presented here suggest that ICI therapy is a promising neoadjuvant treatment that might allow radical excision of borderline resectable or locally advanced dMMR/ MSI-high colorectal cancer, even when the tumor invades other organs. Some previous reports discussed cT4b cases with dMMR/ MSI-high colorectal cancer, but those reported cases showed no FDG accumulation at the tumor site after immunotherapy [24–26], whereas our two cases both showed remnant accumulation of FDG on PET–CT scan after immunotherapy. It is known that FDG uptake can also be induced by some inflammation [27, 28]. Colonoscopy revealed ulcer formation at the tumor site in Case 1 and a fistula in Case 2, which were thought to be results of the response induced by ICI therapy. PET–CT was performed within three weeks after the last administration of ICI therapy, but the degree of FDG accumulation in Case 2 was lower than in Case 1. The duration of neoadjuvant ICI therapy was different: three months in Case 1 and six months in Case 2. It is well known that PET–CT for the assessment of pathological response in locally advanced rectal cancer following neoadjuvant chemoradiation is useful [29–32]. Interestingly, Ciara et al. [33] suggested that early assessment of PET–CT after neoadjuvant chemoradiation for locally advanced rectal cancer failed to show predictive utility. Meanwhile, later assessments of PET–CT following chemoradiation completion showed significantly decreased FDG uptake, which could be a predictive biomarker of a pCR. Similarly, it is likely that no or little FDG uptake may be shown if we assessed the tumor site a few months later in our cases. However, it is still unclear when we should assess PET–CT as a surrogate biomarker of the pCR in borderline resectable or locally advanced dMMR/ MSI-high colorectal cancer treated with neoadjuvant ICI therapy.

Colonoscopy in Case 1 and Case 2 revealed an ulcer and a fistula at each tumor site, respectively, thought to be induced by ICI therapy. However, there was no manifestation of an inflammatory reaction in blood tests just before surgery. In the clinicopathological assessment, we found no fibrosis in the resected tumor that had shown a pCR to ICI therapy. Chemoradiation typically results in remarkable fibrosis on necrosis of the tumor. Yet, in both Case 1 and Case 2, the tumor sites showed primarily the mucus layer, with no viable tumor cells and without disrupting the lesions’ shape.

The JSCCR 2019 guidelines (revised in 2022) recommend immunotherapy as a second line treatment after failure of standard chemotherapy, such as mFOLFOX6 [23]. For this reason, we started with mFOLFOX6 treatment for the patient presented as Case 1. The cytotoxic chemotherapy failed to reduce the volume of tumor. However, subsequent ICI therapy was highly effective in shrinking the tumor size. Current evidence suggests that dMMR/ MSI-high tumors are resistant to conventional chemotherapy, mainly due to the inability of the cells to detect mismatched and unpaired bases [11, 13, 34]. On the other hand, combination therapy consisting of concurrent chemoradiation and surgery is a mainstay of treatment for locally advanced rectal cancer [35, 36]. However, a recent retrospective study using the National Cancer Database showed that microsatellite instability was independently associated with a reduced possibility of pCR after neoadjuvant chemoradiation in patients with locally advanced rectal cancer [37]. The overall pCR rate was 8.6%, including 8.9% for pMMR tumors and 5.9% for dMMR tumors.

Recently, two prospective phase 2 trials showed that neoadjuvant ICI therapy for locally advanced colorectal cancer resulted in high pCR rates [7, 38]. In these studies, the overall pCR rate was 100% for 16 patients with dMMR rectal cancer who were treated with dostarlimab (500 mg) every three weeks for six cycles, and 69% for 30 patients with locally advanced dMMR and 30 patients with pMMR colon cancer who received single dose ipilimumab (1 mg/kg) and two doses of nivolumab (3 mg/kg) every four weeks for one cycle (NICHE trial). These results show the potential of neoadjuvant ICI therapy and suggest it might be more beneficial than cytotoxic chemotherapy or chemoradiation therapy for patients with dMMR/ MSI-h colorectal cancer in terms of overall and/or disease-specific survival.

Interestingly, dostarlimab was administrated for six months in the first study, while in the NICHE trial, treatment was restricted to one cycle for one month. Our patient in Case 1 required combination therapy of ipilimumab and nivolumab for four cycles over three months to achieve sufficient tumor shrinkage resulting in a pCR, but the main tumor site of rectal cancer turned ulcer. Conversely, our patient described as Case 2 received single agent pembrolizumab for eight cycles over six months, resulting in a pCR. The tumor in Case 2 turned fistula which was partially regenerated to normal tissue. It remains unknown what duration of neoadjuvant ICI therapy is appropriate to achieve pCR in locally advanced dMMR/ MSI-h colorectal cancer. In the dostarlimab trial in dMMR/ MSI-h locally advanced rectal cancer, endoscopic biopsies were performed at baseline and during visual inspection of the tumor response at six weeks, three months, and six months, and thereafter every four months. Patients who had a clinical complete response after six months of dostarlimab therapy and had tissue that could be evaluated, did not have evidence of tumor in the endoscopic biopsy, with a majority of patients having no evidence of viable tumor as early as six weeks [7]. There is no evidence for optimal duration of ICI therapy and no biomarkers have been described that can predict complete response in this early period after the start of treatment. Some reports discuss a “watch-and-wait” strategy for patients with clinical complete response of rectal cancer after conventional chemotherapy, and the NCCN guideline also refers to it [39–47]. However, this strategy remains controversial and is challenging for patients, such as Case 1, showing a positive result from imaging studies (PET–CT in Case 1) and for a patient like Case 2 with colon cancer for whom viability of the tumor cannot be assessed by digital examination. Further study is needed to investigate adequate duration of ICI agents before surgery and to determine what factors should be taken into account to allow decisions for the watch-and-wait strategy.

## Conclusions

We presented two cases with locally advanced dMMR/ MSI-h colorectal cancer who underwent R0 resection after a significant response to neoadjuvant ICI therapy. In conclusion, neoadjuvant ICI therapy in locally advanced dMMR/ MSI-h colorectal cancer may be a neoadjuvant option to achieve complete response. Furthermore, neoadjuvant ICI therapy in MMR/ MSI-h colorectal cancer may be able to replace cytotoxic chemotherapy and surgery or diminish the optimal extent of resection.

## Data Availability

The datasets used and/or analyzed during the current study are available from the corresponding author on reasonable request.

## Abbreviations

ICI: Immune checkpoint inhibitors
dMMR: Deficient mismatch repair
MSI-h: Microsatellite instability-high
pCR: Pathological complete response
PD-1: Programmed cell death 1
PD-L1: Programmed cell death 1-ligand 1
NCCN: National Comprehensive Cancer Network
ESMO: European Society for Medical Oncology
JSCCR: The Japanese Society for Cancer of the Colon and Rectum
FDG: Fluorine-18-fluorodeoxy-d-glucose
PET: Positron emission tomography
MRI: Magnetic resonance imaging
CT: Computerized tomography
